# An integrated nomogram combining deep learning, clinical characteristics and ultrasound features for predicting central lymph node metastasis in papillary thyroid cancer: A multicenter study

**DOI:** 10.3389/fendo.2023.964074

**Published:** 2023-02-21

**Authors:** Luchen Chang, Yanqiu Zhang, Jialin Zhu, Linfei Hu, Xiaoqing Wang, Haozhi Zhang, Qing Gu, Xiaoyu Chen, Sheng Zhang, Ming Gao, Xi Wei

**Affiliations:** ^1^Department of Diagnostic and Therapeutic Ultrasonography, Tianjin Medical University Cancer Institute and Hospital, National Clinical Research Center for Cancer, Key Laboratory of Cancer Prevention and Therapy, Tianjin’s Clinical Research Center for Cancer, Tianjin, China; ^2^Department of Thyroid and Neck Cancer, Tianjin Medical University Cancer Institute and Hospital, National Clinical Research Center of Cancer, Key Laboratory of Cancer Prevention and Therapy, Tianjin’s Clinical Research Center for Cancer, Tianjin, China; ^3^Department of Ultrasonography, Cangzhou Hospital of Integrated Traditional Chinese and Western Medicine of Hebei Province, Cangzhou, Hebei, China; ^4^Department of Breast and Thyroid Surgery, Tianjin Union Medical Center, Tianjin, China

**Keywords:** deep learning, papillary thyroid carcinoma, central lymph node metastasis, nomogram, ultrasound

## Abstract

**Objective:**

Central lymph node metastasis (CLNM) is a predictor of poor prognosis for papillary thyroid carcinoma (PTC) patients. The options for surgeon operation or follow-up depend on the state of CLNM while accurate prediction is a challenge for radiologists. The present study aimed to develop and validate an effective preoperative nomogram combining deep learning, clinical characteristics and ultrasound features for predicting CLNM.

**Materials and methods:**

In this study, 3359 PTC patients who had undergone total thyroidectomy or thyroid lobectomy from two medical centers were enrolled. The patients were divided into three datasets for training, internal validation and external validation. We constructed an integrated nomogram combining deep learning, clinical characteristics and ultrasound features using multivariable logistic regression to predict CLNM in PTC patients.

**Results:**

Multivariate analysis indicated that the AI model-predicted value, multiple, position, microcalcification, abutment/perimeter ratio and US-reported LN status were independent risk factors predicting CLNM. The area under the curve (AUC) for the nomogram to predict CLNM was 0.812 (95% CI, 0.794-0.830) in the training cohort, 0.809 (95% CI, 0.780-0.837) in the internal validation cohort and 0.829(95%CI, 0.785-0.872) in the external validation cohort. Based on the analysis of the decision curve, our integrated nomogram was superior to other models in terms of clinical predictive ability.

**Conclusion:**

Our proposed thyroid cancer lymph node metastasis nomogram shows favorable predictive value to assist surgeons in making appropriate surgical decisions in PTC treatment.

## Introduction

The global prevalence of thyroid cancer has sharply increased in the past few decades, and it is also increasing in China. Papillary thyroid carcinoma (PTC) is the most common type of thyroid cancer, which accounts for approximately 80% of all thyroid carcinomas (1–3). PTC is usually an indolent cancer, and the 10-year survival rate of PTC can reach 93% if standardized treatment is accepted (4). However, PTC easily metastasizes to cervical lymph nodes, and the prevalence of central lymph node metastasis (CLNM) can reach as high as 40% to 60% (5, 6). CLNM status is an important risk factor for high recurrence rates and low patient survival (7, 8). A key and controversial problem in thyroid cancer management is if prophylactic central lymph node dissection (CLND) is necessary. CLND is recommended for patients who are suspected of CLNM in preoperative assessment. Some researchers demonstrated that prophylactic CNLD can more accurately stage tumors and reduce recurrence rates in patients with intermediate and high-risk thyroid cancer, while others argue that patients may gain no benefit or some temporary morbidity (such as hypocalcemia and spinal accessory nerve dysfunction), and endoscopic lymphadenopathy (9, 10). Therefore, most studies do not recommend the routine use of prophylactic CLND in PTC (11, 12).

According to the American Thyroid Association guidelines, preoperative neck ultrasound (US) is recommended to evaluate cervical lymph nodes, but the diagnosis rate is not accurate; although it has high diagnostic value for accessing lateral lymph node metastasis(LLNM), it has relatively low sensitivity in the diagnosis of CLNM (13–15).Thus, an effective and non-invasive way to predict CLNM before surgery is urgently needed to provide optimal surgical treatments.

Thyroid cancer nomograms are widely used as a prognostic tool to understand the nature of thyroid cancer lesions, assess the unknown risk of disease and predict the possible outcome of treatment. Several established models based on the clinical and ultrasound characteristics to predict CLNM, but their performance is not satisfactory and not adaptable to actual clinical work (16–19). Currently, the use of artificial intelligence (AI) in medicine has gained interest, particularly in analyzing and diagnosing medical images (20, 21). AI models provide another opinion to assist radiologists in interpreting the images by improving the accuracy and consistency of disease diagnosis and by reducing the time to output results. Several studies have investigated deep learning to diagnose thyroid malignancy and have achieved better performance than human readers (22–25). Therefore, the prospect of deep learning in predicting lymph node metastasis is worth exploring. To our knowledge, deep learning has not yet been integrated with clinical and ultrasound factors to construct a combined nomogram to predict CLNM in PTC patients.

**Figure 1:**
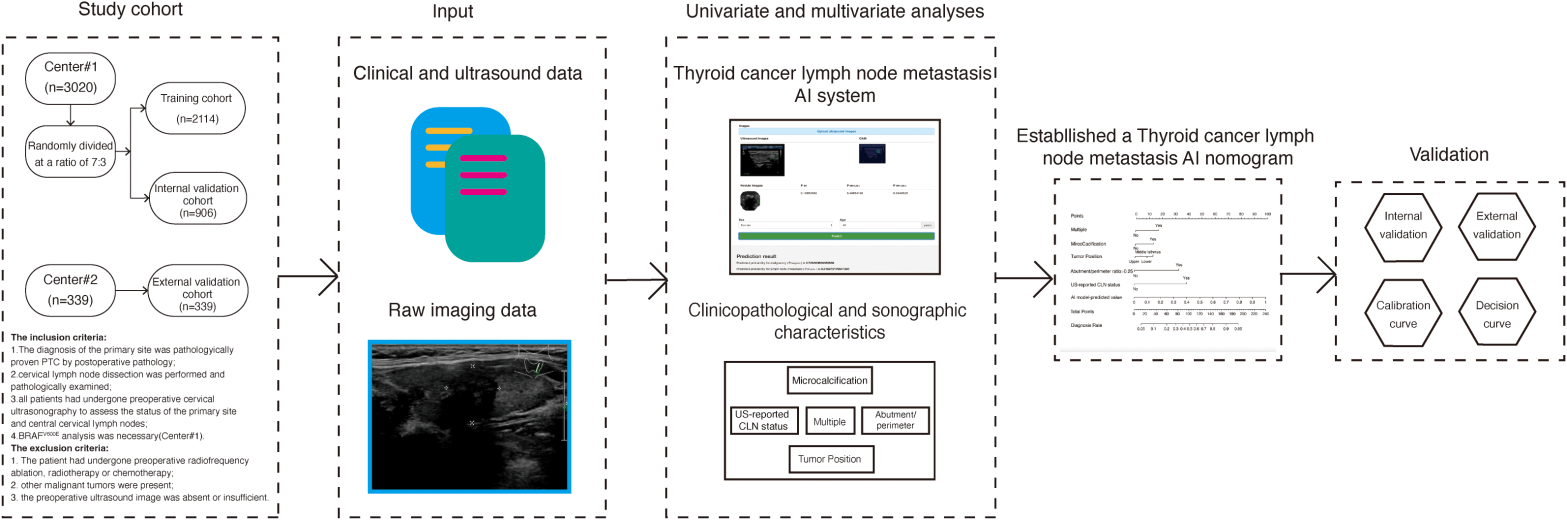
Flow diagram of the thyroid cancer lymph node metastasis nomogram.

Recently, we developed a thyroid cancer lymph node metastasis AI system-based deep learning model to predict the status of cervical lymph nodes. The purpose of the present study was to (1) evaluate the value of this system to predict CLNM and to (2) combine it with additional clinical and ultrasound characteristics to establish a more robust and generalizable model for the prediction of CLNM in PTC patients. The flowchart of our model development is shown in **Figure 1**.

## Materials and methods

### Patients

In this study, a total of 3359 PTC patients who had undergone first-time thyroidectomy to treat thyroid carcinoma at two centers (center#1: Tianjin Medical University Cancer Institute and Hospital; center#2: Binzhou Medical University Hospital) were enrolled from March 2011 to June 2018. Data from center#1 were randomly divided into two groups as follows: 70% for a training cohort (n=2114) and 30% for an internal validation cohort(n=906), respectively. Data from center#2 (n = 339) were used for an external validation cohort. This study was approved by the Ethics Committee of Tianjin Medical University Cancer Institute and Hospital(No. bc2020190), and the requirements for informed consent were waived.

Total thyroidectomy or thyroid lobectomy with therapeutic or prophylactic lymph node dissection was performed for patients. Ipsilateral central neck lymph node dissection (CLND) was routinely performed, total thyroidectomy with bilateral CLND was performed for patients with bilateral PTC or patients with clinical evidence of contralateral CLNM. At the center#1, the inclusion criteria were as follows: (1) the diagnosis of the primary site was pathologically proven PTC by postoperative pathology; (2) cervical lymph node dissection was performed and pathologically examined; (3) all patients had undergone preoperative cervical ultrasonography to assess the status of the primary site and central cervical lymph nodes; and (4) BRAF^V600E^ analysis was necessary. The exclusion criteria were as follows: (1) the patient had undergone preoperative radiofrequency ablation, radiotherapy or chemotherapy; (2) other malignant tumors were present; and (3) the preoperative ultrasound image was absent or insufficient. All pathology specimens were reviewed retrospectively by two or more experienced pathologists. At the center#2, we have the same inclusion criteria, but the BRAF^V600E^ analysis is not within the scope of the record.

### Ultrasonography and image analysis

All patients had undergone ultrasound examination before surgery to assess the characteristics of the nodules and status of the central lymph node (CLN). All ultrasound images were reviewed and interpreted by two experienced radiologists (Dr. Wei X and Dr. Zhang S with 15 and 30 years of experience in thyroid cancer US diagnosis, respectively).

All ultrasound examinations used a Phillips EPIQ 5, IU 22, HD11 (Philips Healthcare, Eindhoven, The Netherlands) equipped with a high-frequency linear array probe (5–12 MHz) to perform thyroid cross-section, longitudinal section and cervical lymph node scan. We reviewed the size, number, location and sonographic features of the thyroid lesions in the longitudinal and transverse axes. The size of the tumor was the largest diameter of the tumor and was divided into the following groups: ≤0.5 cm, 0.5~1.0 cm, 1.0~2.0 cm and ≥2.0 cm. If there was more than one nodule that was suspected as malignant, we defined it as multiple. In multiple cases, the size of the tumor was classified according to the diameter of the largest tumor. The position of the lesion was evaluated from the following four aspects: upper, middle, lower and isthmus. We further divided several typical ultrasound features of nodules according to the American College of Radiology Thyroid Imaging Reporting and Data System (ACR TI-RADS) (26), including the composition (mixed cystic and solid or solid), echogenicity (isoechoic or hyperechoic, hypoechoic or very hypoechoic), shape (taller-than-wide<1 or taller-than-wide≥1), margin (clear or unclear) and microcalcifications (yes or no). In addition, Hashimoto’s thyroiditis and the abutment of the thyroid capsule were evaluated on the basis of ultrasound images. Hashimoto’s thyroiditis manifests as uneven echogenicity of the thyroid parenchyma on ultrasonography, with a few or multiple lamellar hypoechoic areas showing grid-like changes. The measurement of abutment or the perimeter in a thyroid lesion was calculated by the average ratio (1/2) on the transverse + longitudinal section of a nodule. Based on our previous study (27), we selected the 1/4 (25%) perimeter of the thyroid lesion as the cutoff value.

The ultrasound features of the involved lymph nodes were based on various criteria. Lymph nodes showing one or more suspicious US features (internal microcalcifications, cystic changes, hyperechogenicity, round shape, loss of hilar echogenicity or the presence of peripheral flow) on ultrasound images were regarded as US-reported metastatic CLN according to ACR TI-RADS (26).

### BRAF^V600E^ mutation analysis

Fine needle aspiration biopsies were repeated at least three times per aspiration using a 22-gauge needle for BRAF^V600E^ mutation assessment in cytology. Immediately after aspiration, the needle and syringe were washed with 1 ml of normal saline, and samples with sufficient numbers of tumor cells were used for real-time PCR analysis (28).

### Thyroid cancer lymph node metastasis AI system

The fully automated deep learning model used in this research was developed by the author, ZHZ, and a cloud-based artificial intelligence diagnosis platform, named the thyroid cancer lymph node metastasis AI system, was established based on this model. This model uses the Mask R-CNN framework as a computer vision framework for nodules segmentation and has the following capabilities: uses a convolutional neural network as a backbone; automatically learns and trains according to the range of artificially labeled nodules; recognizes the relevant features of the nodule; and uses the relevant features as quantifiable parameters (29). The nodule image was then enlarged to 1.5 times the pixel size, and the SE-ResNeXt-50 model was used as the classification model (30). We used mirrored, rotated, folded and normalized data augmentation during the training process and applied random dropout to prevent the model from overfitting. ResNeXt improves the accuracy without increasing the complexity of the parameters while also reducing the number of hyperparameters. After predicting each image of the patient’s nodule by the SE-ResNeXt-50 neural network, we obtained the corresponding labels [benign nodules (BN), malignant nodules without cervical lymph node metastasis (MN-LN(-)), and malignant nodules with cervical lymph node metastasis (MN-LN(+))] of the patient. A logistic regression model was then established based on the relationships among cervical lymph node status, sex, age and model prediction results. Finally, we obtained the final AI model-predicted value of the patient’s probability of cervical lymph node metastasis. The cloud-based AI diagnosis platform can be accessed on the following website: http://thyai.zzinf.com/.

Our proposed model was implemented using Python (version 3.7.6; https://www.python.org/), DL toolkit Pytorch (version 1.5.1; https://pytorch.org/) and OpenCV (version 4.4.0; https://opencv.org). All experiments were conducted on a workstation equipped with a NVDIA 2080Ti GPU.

### Development of a thyroid cancer lymph node metastasis nomogram

We used data in the training cohort to construct the nomogram, which included the AI-predicted value, clinical characteristics and ultrasound features. Pearson’s chi-squared test and t-test were applied for univariate analysis. Statistically significant factors in univariate analysis were incorporated into multivariate logistic regression analysis to construct the predictive model. Statistical significance was decided by a criterion of two-sided P<0.05. A nomogram was then developed based on the multivariate analysis in the training cohort.

### Model evaluation and comparison

To fully evaluate our thyroid cancer lymph node metastasis nomogram, we compared the model with the following other methods: (1) the thyroid cancer lymph node metastasis AI system; (2) a clinical model based on the age, sex and remaining ultrasound features of the primary site in the multiple logistic regression analysis; and (3) the US-reported CLN status based on the involved lymph nodes.

### Statistical analysis

The Mann-Whitney U test and chi-square test were separately used to compare the differences in continuous variables and categorical variables. The model predictions were assessed by sensitivity, specificity, positive predictive value, negative predictive value, the area under the curve (AUC) of the receiver operating characteristic (ROC) curve and 95% confidence interval (CI) as well as calibration curves in both the training and validation cohorts. Delong test was used to compare different AUC. Calibration plot analysis was performed by bootstrapping with 1,000 replications. Decision curve analysis was used to evaluate clinical usefulness and net benefits. All analyses were performed using R statistical software (version 3.3.3; www.R-project.org). P<0.05 was considered statistically significant.

## Results

### Clinical characteristics and ultrasound features of the patients

The clinical and ultrasound characteristics of the patient are summarized in **Table 1**.

**Table 1:** Clinicopathological and sonographic characteristics of patients in PTC by central lymph node status.

	Training cohort n=2114			Internal Validation cohort n=906			External Validation cohort n=339		
Characteristic	CLNM (+)	CLNM (-)	P value	CLNM (+)	CLNM (-)	P value	CLNM (+)	CLNM (-)	P value
Gender			**<0.001**			**0.04**			0.06
Female	746(70.2%)	851(81%)		340(73.9%)	356(79.8%)		116(71.6%)	143(80.8%)	
Male	317(29.8%)	200(19%)		120(26.1%)	90(20.2%)		46(28.4%)	34(19.2%)	
Age (years)			**<0.001**			**<0.001**			0.116
>55	114(10.7%)	234(22.3%)		36(7.8%)	106(23.8%)		28(17.3%)	44(24.9%)	
≤55	949(89.3%)	817(77.7%)		424(92.2%)	340(76.2%)		134(82.7%)	133(75.1%)	
Braf ^V600E^ mutation			0.250			0.743			–
No	212(19.9%)	188(17.9%)		100(21.7%)	92(20.6%)		/	/	
Yes	851(80.1%)	863(82.1%)		360(78.3%)	354(79.4%)		/	/	
Position			**<0.001**			0.337			0.133
Upper	281(26.4%)	365(34.7%)		145(31.5%)	156(35%)		20(12.3%)	21(11.9%)	
Middle	288(27.1%)	251(23.9%)		98(21.3%)	105(23.5%)		87(53.7%)	84(47.5%)	
Lower	454(42.7%)	399(38.0%)		198(43%)	172(38.6%)		46(28.4%)	68(38.4%)	
Isthmus	40(3.8%)	36(3.4%)		19(4.2%)	13(2.9%)		9(5.6%)	4(2.3%)	
Diameter			**<0.001**			**<0.001**			**<0.001**
≤0.5	7(0.6%)	24(2.3%)		5(1.1%)	11(2.5%)		13(8%)	42(23.7%)	
0.5-1	244(23.0%)	424(40.3%)		110(23.9%)	167(37.4%)		57(35.2%)	104(58.8%)	
1-2	555(52.2%)	495(47.1%)		246(53.5%)	219(49.1%)		62(38.3%)	22(12.4%)	
≥2	257(24.2%)	108(10.3%)		99(21.5%)	49(11%)		30(18.5%)	9(5.1%)	
Microcalcification			**<0.001**			**<0.001**			**0.001**
No	118(11.1%)	282(26.8%)		66(14.3%)	119(26.7%)		31(19.1%)	63(35.6%)	
Yes	945(80.9%)	769(73.2%)		394(85.7%)	327(73.3%)		131(80.9%)	114(64.4%)	
Margin			0.791			0.633			1
Clear	78(7.3%)	73(6.9%)		39(8.5%)	33(7.4%)		11(6.8%)	13(7.3%)	
unclear	985(92.7%)	978(93.1%)		421(91.5%)	413(92.6%)		151(93.2%)	164(92.7%)	
Shape			**0.012**			0.249			0.095
Taller-than-wide < 1	350(32.9%)	292(27.8%)		145(31.5%)	124(27.8%)		34(21%)	24(13.6%)	
Taller-than-wide≥1	713(67.1%)	759(72.2%)		315(68.5%)	322(72.2%)		128(79%)	153(86.4%)	
Composition			0.68			0.579			1
Mixed cystic and solid	12(1.1%)	9(0.9%)		7(1.5%)	4(0.9%)		4(2.5%)	5(2.8%)	
Solid	1051(98.9%)	1042(99.1%)		453(98.5%)	442(99.1%)		158(97.5%)	172(97.2%)	
Echo			**<0.001**			**0.026**			0.340
Hyper or isoechoic	20(1.8%)	18(1.7%)		9(2%)	7(1.6%)		14(8.6%)	9(5.1%)	
Hypo-echoic	818(77.0%)	729(69.4%)		347(75.4%)	303(67.9%)		120(74.1%)	131(74%)	
Very Hypo-echoic	225(21.2%)	304(28.9%)		104(22.6%)	136(30.5%)		28(17.3%)	37(20.9%)	
Abutment/perimeter			**<0.001**			**<0.001**			**<0.001**
≤ 0.25	295(27.8%)	665(63.3%)		119(25.9%)	270(60.5%)		53(32.7%)	143(80.8%)	
>0.25	768(72.2%)	386(36.7%)		341(74.1%)	176(39.5%)		109(67.3%)	34(19.2%)	
Hashimoto thyroiditis			0.257			0.329			**<0.001**
Negative	845(79.5%)	857(81.5%)		371(80.7%)	347(77.8%)		103(63.6%)	162(91.5%)	
Positive	218(20.5%)	194(18.5%)		89(19.3%)	99(22.2%)		59(36.4%)	15(8.5%)	
Multiple			**<0.001**			**<0.001**			0.533
Negative	595(56%)	719(68.4%)		239(52%)	315(70.6%)		99(61.1%)	115(65%)	
Positive	468(44%)	332(31.6%)		221(48%)	141(29.4%)		63(38.9%)	62(35%)	
US-reported CLN status			**<0.001**			**<0.001**			**<0.001**
Negative	642(60.4%)	956(91%)		291(63.3%)	412(92.4%)		103(63.6%)	162(91.5%)	
Positive	421(39.6%)	95(9%)		169(36.7%)	34(7.6%)		59(36.4%)	15(8.5%)	
AI model predicted value			**<0.001**			**<0.001**			**<0.001**
median(interquartile range)	0.58(0.45-0.76)	0.42(0.29-0.53)		0.58(0.45-0.74)	0.42(0.29-0.54)		0.38(0.26-0.56)	0.27(0.17-0.39)	

Bold values indicate statistical significance (p < 0.05).

### Univariate and multivariate analyses of CLNM risk factors

**Table 1** shows the association between CLNM and several risk factors in PTC patients. According to univariate analysis, gender (P<0.001), age (P<0.001), tumor size (P<0.001), tumor position (P<0.001), multiple (P<0.001), taller-than-wide (P=0.012), echo (P<0.001), microcalcification (P<0.001), abutment/perimeter ratio>0.25 (P<0.001), US-reported CLN status (P<0.001) and AI model-predicted value (P<0.001) were closely related to CLNM. Multivariate regression analysis showed that AI model-predicted value (P<0.001), tumor position (P<0.001), multifocality (P<0.001), microcalcification (P=0.001), abutment/perimeter ratio>0.25 (P<0.001) and US-reported CLN status (P<0.001) were significantly associated with a high risk of CLNM (**Table 2**).

**Table 2:** Risk factors for central lymph node metastasis in the training cohort.

Clinical variable	Multivariate analysis			
	Estimate	Std. Error	95%CI	P value
Diameter				
≤0.5	–	–	–	–
0.5-1	0.162	0.463	0.497-3.125	0.726
1-2	0.071	0.463	0.453-2.855	0.878
≥2	0.240	0.482	0.515-3.490	0.619
Position				
Upper	–	–	–	–
Middle	0.648	0.142	1.449-2.527	**<0.001**
Lower	0.506	0.127	1.293-2.130	**<0.001**
Isthmus	-0.141	0.295	0.487-1.550	0.633
Microcalcification				
No	–	–	–	–
Yes	0.457	0.141	1.201-2.085	**0.001**
Shape				
Taller-than-wide < 1	–	–	–	–
Taller-than-wide≥1	0.520	0.122	0.829-1.340	0.671
Echo				
Hyper or isoechoic	–	–	–	–
Hypo-echoic	0.351	0.385	0.667-3.041	0.363
Very Hypo-echoic	0.252	0.396	0.592-2.813	0.524
Abutment/perimeter				
≤ 0.25	–	–	–	–
>0.25	1.166	0.111	2.582-3.995	**<0.001**
Multiple				
Negative	–	–	–	–
Positive	0.638	0.109	1.530-2.345	**<0.001**
US-reported CLN status				
Negative	–			
Positive	1.311	0.140	2.828-4.898	**<0.001**
AI model predicted value	3.397	0.283	17.264-52.341	**<0.001**

Bold values indicate statistical significance (p < 0.05).

### Construction and validation of the thyroid cancer lymph node metastasis nomogram

**Figure 2:**
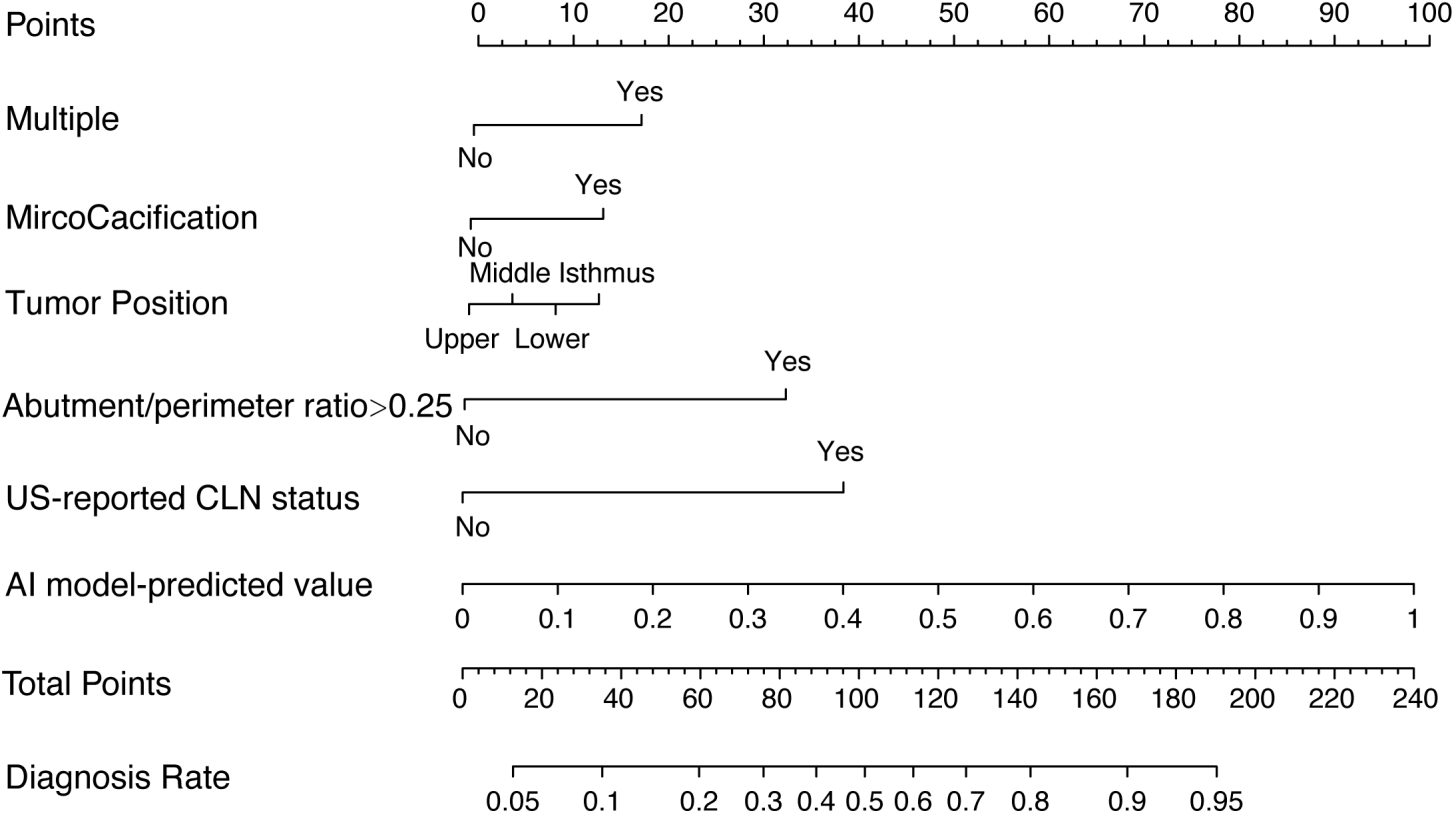
Thyroid cancer lymph node metastasis nomogram for predicting CLNM in PTC patients.

**Figure 3:**
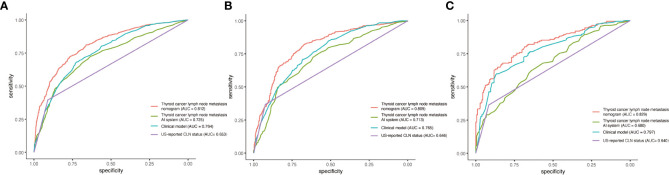
Performance of the thyroid cancer lymph node metastasis nomogram, thyroid cancer lymph node metastasis AI system, clinical model and US-reported CLN status in PTC patients. **(A)** ROC curve for predicting CLNM in the training cohort. **(B)** ROC curve for predicting CLNM in the internal validation cohort. **(C)** ROC curve for predicting CLNM in the external validation cohort.

**Figure 4:**
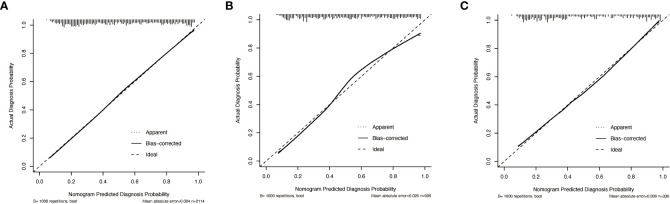
Calibration curves of the thyroid cancer lymph node metastasis nomogram, thyroid cancer lymph node metastasis AI system, clinical model and US-reported CLN status in PTC patients. **(A)** Calibration curves for predicting CLNM in the training cohort. **(B)** Calibration curves for predicting CLNM in the internal validation cohort. **(C)** Calibration curves for predicting CLNM in the external validation cohort.

Multivariable analysis demonstrated that the AI model-predicted value, multifocality, tumor position, microcalcification, abutment/perimeter ratio and US-reported CLN status remained important predictors of CLNM metastasis in PTC patients. Thus, a thyroid cancer lymph node metastasis nomogram incorporating these six predictors was constructed (**Figure 2**). The nomogram showed that the AI model-predicted value was the largest contributor to the scores followed by US-reported CLN status and abutment/perimeter ratio>0.25. Microcalcification, tumor position and multifocality showed a modest impact on the model. The AUC for the nomogram to predict CLNM was 0.812 (95% CI, 0.794-0.830) in the training cohort, 0.809 (95% CI, 0.780-0.837) in the internal validation cohort, and 0.829 (95%CI, 0.785-0.872) in the external validation cohort (**Figure 3A–C**). The calibration curve of this nomogram presented good agreement between the bias-corrected prediction and ideal reference line with an additional 1000 bootstraps in the training and two validation cohorts (**Figure 4A–C**).

### Performance of the thyroid cancer lymph node metastasis nomogram

We also compared the thyroid cancer lymph node metastasis nomogram with the thyroid cancer lymph node metastasis AI system, clinical model and US-reported CLN status. In the training cohort, the nomogram yielded remarkable performance (sensitivity = 72.4%, specificity = 76.5% and AUC = 0.812; 95% CI: 0.794-0.830) and was superior to the thyroid cancer lymph node metastasis AI system (sensitivity = 67.3%, specificity = 68.9% and AUC = 0.725; 95% CI: 0.703-0.746), clinical model (sensitivity =68.9%, specificity = 72.1% and AUC = 0.764; 95% CI: 0.744-0.784) and US-reported CLN status (sensitivity =39.6%, specificity = 91.0% and AUC = 0.653; 95% CI: 0.636-0.670). In the internal validation cohort, the performance of the nomogram also achieved reasonable performance (sensitivity = 73.9%, specificity = 76.7% and AUC = 0.809; 95% CI: 0.780-0.837) and was better than that of the thyroid cancer lymph node metastasis AI system (sensitivity =50.9%, specificity = 83.2% and AUC = 0.713; 95% CI: 0.679-0.746), clinical model (sensitivity =78.1%, specificity = 60.5%, and AUC = 0.765; 95% CI: 0.734-0.795) and US-reported CLN status (sensitivity =36.7%, specificity = 92.4%, and AUC = 0.646; 95% CI: 0.620-0.671). In the external validation cohort, the thyroid cancer lymph node metastasis nomogram still shows the most outstanding performance, compared with several other models. (**Table 3**)

**Table 3:** Identification performance of thyroid cancer lymph node metastasis nomogram, thyroid cancer lymph node metastasis AI system, clinical model and US-reported CLN status in the training cohort, internal validation cohort and external validation cohort.

	Training cohort				Internal Validation cohort				External Validation cohort			
Model	Thyroid cancer lymph node metastasis nomogram	Thyroid cancer lymph node metastasis AI system	Clinical model	US-reported CLN status	Thyroid cancer lymph node metastasis nomogram	Thyroid cancer lymph node metastasis AI system	Clinical model	US-reported CLN status	Thyroid cancer lymph node metastasis nomogram	Thyroid cancer lymph node metastasis AI system	Clinical model	US-reported CLN status
AUC (95% CI)	0.812 (0.794-0.830)	0.725 (0.703-0.746)	0.764 (0.744-0.784)	0.653 (0.636-0.670)	0.809 (0.780-0.837)	0.713 (0.679-0.746)	0.765 (0.734-0.795)	0.646 (0.620-0.671)	0.829 (0.785-0.872)	0.680 (0.624-0.737)	0.797 (0.749-0.845)	0.640 (0.597-0.682)
Sensitivity	0.724	0.673	0.689	0.396	0.739	0.509	0.781	0.367	0.759	0.630	0.654	0.364
Specificity	0.765	0.689	0.721	0.910	0.767	0.832	0.605	0.924	0.768	0.638	0.859	0.915
Positive predictive value	0.757	0.686	0.714	0.816	0.766	0.757	0.656	0.833	0.750	0.614	0.809	0.797
Negative predictive value	0.733	0.675	0.696	0.598	0.740	0.621	0.742	0.586	0.777	0.653	0.731	0.611
P value	-	**<0.001**	**<0.001**	**<0.001**	-	**<0.001**	**<0.001**	**<0.001**	-	**<0.001**	**<0.001**	**<0.001**

Significant differences between Thyroid cancer lymph node metastasis nomogram and other models. The DeLong test method was used to compare AUCs. Bold values indicate statistical significance (p < 0.05).

**Figure 5:**
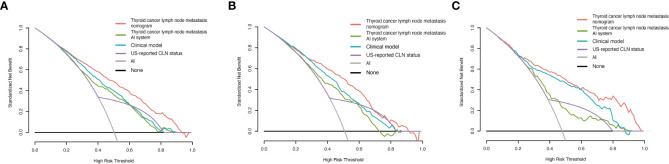
Decision curve analysis of the thyroid cancer lymph node metastasis nomogram, thyroid cancer lymph node metastasis AI system, clinical model and US-reported CLN status in PTC patients. **(A)** Decision curve analysis for predicting CLNM in the training cohort. **(B)** Decision curve analysis for predicting CLNM in the internal validation cohort. **(C)** Decision curve analysis for predicting CLNM in the external validation cohort.

We also performed decision curve analysis (DCA) to compare the clinical usability and benefits of predicting the risk of CLNM of this model with those of the traditional ultrasound methods. The DCA curves of the new nomogram showed greater net benefits across a range of CLNMs risks in the three cohorts than the other models (**Figure 5A–C**).

## Discussion

Lymph node metastasis is common among PTC patients. Thyroidectomy combined with therapeutic lymph node dissection has become a preferred initial surgical strategy for PTC patients with clinically positive lymph nodes, but disagreement persists regarding whether patients with negative lymph node metastasis on ultrasound should undergo preventive central neck dissection. Total thyroidectomy plus prophylactic CLND and total thyroidectomy alone did not differ significantly in local recurrence rates or postoperative complication rates between PTC patients with clinically node-negative. Therefore, prophylactic CLND may not be required if total thyroidectomy is planned (31, 32).

Ultrasonography is one of the most commonly used non-invasive tools applied to the examination of thyroid nodules, but it still has some limitations. For example, ultrasonography relies greatly on the radiologists’ work experience, and less experienced radiologists may show less accuracy in the identification of CLNM. Therefore, it is necessary to develop a reliable and noninvasive preoperative tool to predict CLNM in PTC.

In the present study, we performed a systematic and comprehensive analysis of the clinical and ultrasound characteristics of 3359 patients from two medical centers with PTC and incorporated our established thyroid cancer lymph node metastasis AI system. We then developed and validated a thyroid cancer lymph node metastasis nomogram to predict CLNM. The AUC value of our model was exceeded 80% in both the training and two validation cohorts. Additionally, the calibration plot showed that the predicted and observed metastasis risks of CLNM were in good agreement. Thus, our model has good practical effects in clinical applications.

Nomograms are widely used for the prediction of cancer prognosis, mainly for their ability to simplify statistical prediction models to estimate the probability of occurrence of an event (e.g., death or recurrence) and to make predictions based on the actual situation of individual patients (33). In general, one of the most conventional methods for predicting lymph node metastasis in PTC is to establish a clinical model that integrates the statistical analysis information of ultrasound and clinicopathological factors. For instance, Wang et al. established a nomogram to predict CLNM in PTMC patients and considered that age < 55 years, male sex, tumor size 0.5-1.0 cm, multifocal lesions, extrathyroidal extension and lateral lymph node metastasis are independent risk factors for CLNM (34). Lu et al. screened six variables for demographic and clinicopathological characteristics as potential risk factors and further constructed a model for lymph node involvement based on the Surveillance, Epidemiology, and End Results (SEER) Program (35). Although a large amount of data was included in the above studies, the performance of these models was not satisfactory and lacked detailed analysis of several clinical features, such as the absence of BRAF^V600E^ mutation testing.

Deep learning consists of a neural network including many layers and features extracted from the original input image. Currently, deep learning is widely used in medical imaging to achieve computer-aided diagnosis, providing assisted diagnostic suggestions in clinical settings and obtaining more accurate results faster than radiologists. In our previous research, we performed a series of studies on artificial intelligence diagnosis of the thyroid in the area of ultrasound. We applied a large dataset of over 300,000 images to build a DCNN model, which had ameliorative accuracy, sensitivity and specificity in the classification of thyroid nodules. The area under the curve exceeded 90% for both the internal test set and the external test set (36). In addition, we constructed a deep CNN model, named the Brief Efficient Thyroid Network (BETNET), for the localization and classification of thyroid nodules, which precisely and intuitively shows the nodular characteristic area with a higher weight within the neural network (22). The abovementioned studies demonstrate the feasibility of applying a deep convolutional neural network in patients with PTC. Thus, we aimed to establish a deep learning platform for the prediction of lymph node status in patients with PTC before surgery.

There have been a few studies applying deep learning models to predict CLNM from ultrasound images. However, these previous studies are based on traditional machine learning-based radiomics methods that extract intensity, boundary, texture and wavelet features from ultrasound images and establish the relationship between these high-throughput features and lymph node status (37). Jiang et al. established a radiomics nomogram based on the SWE radiomics signature and achieved an area under the curve of nearly 85% (37). Although this model achieved better results, the sample size of the patients was only 237, suggesting that the diagnostic value of the shear-wave elastography images in thyroid ultrasound is debatable (38). Additionally, due to the imbalance of medical development in different regions, this model based on SWE radiomics it is not universal to each hospital.

In our preliminary work, we successfully established a cloud-based artificial intelligence diagnosis platform called the thyroid cancer lymph node metastasis AI system based on deep learning to accurately localize thyroid nodules on ultrasound and automatically distinguish the nature of nodules and lymph node status, which included age and sex as independent risk factors affecting cervical lymph node metastasis. Due to the different scanning angles and positions, the object area may pan, and its shape may change, causing differences between images of the same category. Unlike previous deep learning models that predict classification results from a single image, our system simultaneously inputs multiple images of different sections of a patient’s lesion and precisely visualizes and locates the nodes to determine the status of the lymph nodes in a comprehensive manner, which is highly interpretable and can be better implemented for clinical applications. In this study, we further evaluated the diagnostic performance of this system in assessing the risk of CLNM.

We then performed detailed statistical analysis of the other clinical and ultrasound characteristics of the patients. Ultrasound parameters are descriptive and distinguishable, and they can be used as a reference for ACR classification. Certain independent risk factors in the clinical features also assist in the determination of lymph node metastasis. The results of the present suggested that multifocality, tumor position, microcalcification, abutment/perimeter>0.25, AI-predicted value and US-reported CLN status were significantly associated with CLNM. These independent risk factors were incorporated to form a new combined model, and a nomogram called the thyroid cancer lymph node metastasis nomogram was developed at the same time. In addition, the external validation further proves the universality of our model. Although the diagnostic performance of the thyroid cancer lymph node metastasis AI system is slightly lower than that of clinical model, AI-predicted value as the largest contributor to scores in the nomogram.

According to ACR TI-RADS guidelines, calcifications within thyroid cancer are classified into four types as follows: microcalcifications, macrocalcifications, marginal calcifications and noncalcifications. Many studies have reported that the formation of microcalcifications is caused by the rapid proliferation of cancer cells and is significantly associated with the incidence of CLNM (39). In our previous studies, we evaluated cervical lymph node metastases based on the tumor abutment/perimeter ratio of the primary site, providing a relatively high reference value to assess the risk of papillary thyroid microcarcinoma metastasis (27). As expected, the 1/4 (25%) perimeter of the thyroid lesion was closely related to CLNM in PTC patients. Moreover, in the present study of Chinese patients, we identified a cohort of more than 3000 patients with classic PTC to explore the relationship between BRAF^V600E^ mutation and CLNM. To our knowledge, this is the largest cohort of samples included in relevant research, and we conclude that BRAF^V600E^ has limited value as an indicator of the risk of CLNM in PTC.

The present study had several limitations. First, regardless of training, internal validation or external validation cohort, our model performed well. These results need to be validated with a larger prospective cohort to test the application value of our model in clinical practice. Second, the immunohistochemical staining patterns and results of fine-needle aspiration may affect the prediction of lymph node status. Thus, we should incorporate these factors into a future study. Finally, our model was only applied to PTC and not to other thyroid cancer subtypes. Future research will focus on other thyroid cancer subtypes, including medullary thyroid carcinoma and follicular thyroid carcinoma.

## Conclusion

In the present study, we developed a thyroid cancer lymph node metastasis nomogram combining deep learning, clinical characteristics and ultrasound features. This nomogram will serve as a noninvasive tool to predict CLNM in patients with PTC that will assist inexperienced radiologists in diagnosing the status of lymph nodes and provide effective guidance to surgeons in preoperative diagnosis.

## Data availability statement

The raw data supporting the conclusions of this article will be made available by the authors, without undue reservation.

## Ethics statement

This study was approved by the Ethics Committee of Tianjin Medical University Cancer Institute and Hospital (No. bc2020190), and the requirements for informed consent were waived.

## Author contributions

LC, SZ, MG, and XW designed this study. YZ, JZ, XQW and XC conducted the experiment and interpreted the data. HZ, XC, QG and LH analyzed the data. All authors contributed to the article and approved the submitted version.
